# Evaluation of new generation systemic immune-inflammation markers to predict urine culture growth in urinary tract infection in children

**DOI:** 10.3389/fped.2023.1201368

**Published:** 2023-10-18

**Authors:** Yusuf Elgormus, Omer Okuyan, Seyma Dumur, Ugurcan Sayili, Hafize Uzun

**Affiliations:** ^1^ Clinic of Pediatrics, Medicine Hospital, İstanbul, Türkiye; ^2^ Department of Pediatrics, Medicine Hospital, Istanbul Atlas University, Istanbul, Türkiye; ^3^ Department of Medical Biochemistry, Faculty of Medicine, Istanbul Atlas University, Istanbul, Türkiye; ^4^ Department of Public Health, Cerrahpasa Faculty of Medicine, Istanbul University-Cerrahpasa, Istanbul, Türkiye

**Keywords:** urinary tract infection, children, C-reactive protein, neutrophil–lymphocyte ratio, platelet–lymphocyte ratio, systemic immune-inflammation index

## Abstract

**Objective:**

Systemic inflammation has been implicated in the development and progression of urinary tract infection (UTI). Accordingly, the aim of this study is to determine whether the white blood cell (WBC), C-reactive protein (CRP), neutrophil–lymphocyte ratio (NLR), platelet–lymphocyte ratio (PLR), and systemic immune-inflammation index (SII) are useful markers to predict of urine culture growth in children with UTI. The second aim of this study is to evaluate the prevalence of UTI pathogens, antibiotic resistance patterns, and empirical treatment options in children diagnosed with UTI based on laboratory and clinical findings.

**Method:**

The study population comprised 413 cases (positive urine culture) and 318 cases (negative urine culture) of pediatric patients with UTI.

**Results:**

There was no statistically significant difference observed in the median levels of hemoglobin, hematocrit, and platelet between the negative and positive culture groups. The median levels of monocytes, WBC, NLR, SII, and CRP of the patients with a positive urine culture were shown to be statistically significantly higher than the patients with a negative urine culture. The AUC value was 0.747 (0.710–0.784) for CRP with a cutoff value of 3.2, the sensitivity value was 56.4%, and the specificity value was 98.4% in terms of UTI. The AUC value was 0.733 (0.697–0.769) for SII with a cutoff value of 600, the sensitivity value was 58.4%, and the specificity value was 83.0%. The AUC value was 0.732 (0.697–0.769) for NLR with a cutoff value of 2, the sensitivity value was 57.4%, and the specificity value was 81.1%.

**Conclusion:**

WBC, CRP, NLR, PLR, and SII could potentially serve as useful independent diagnostic or complementary markers for disease in children diagnosed with UTI who exhibit a positive urine culture. *Escherichia coli* was found to be the most common causative agent, and the commonly prescribed antibiotic was cephalosporin. However, it was observed that all identified agents of pediatric UTIs in our center exhibited high resistance to cefuroxime, trimethoprim–sulfamethoxazole, cefixime, ampicillin, and ceftriaxone.

## Introduction

Urinary tract infection (UTI) in children is one of the most common and important issues in the field of nephrology. The term UTI reflects clinical and pathological conditions characterized by bacteriuria, involving various parts of the urinary system. These infections can be symptomatic as well as asymptomatic. In order to be successful in treating recurrent UTI and its complications, an early diagnosis and initiation of pathogenesis-oriented treatment are important. A symptomatic UTI can be classified into two clinical categories: upper UTI (UUTI) or acute pyelonephritis (APN) and lower UTI (LUTI), which affects the lower urinary tract, primarily cystitis (1, 2).

The high incidence rate, propensity for relapse, associated morbidity, and difficulties in obtaining an uncontaminated urine specimen pose significant challenges to clinicians. Collecting an uncontaminated urine specimen is essential for accurate diagnosis. Rapid diagnosis and treatment are important for preventing acute complications as well as renal scarring. The diagnosis of UTI is based on the demonstration of a significant number of bacteriuria in culture taken from urine samples under appropriate conditions [>10^5^ colony-forming units (CFU)/ml] (3). Inappropriate antibiotic and/or dose selection increases the possibility of resistance in UTI (4). However, the specific benefits and harms of such treatments remain unclear (5).

In majority of children with fever, it is possible to determine the fever focus by the complaints and physical examination findings and to determine whether the microorganism causing the fever is bacteria or virus (6). However, in some cases, it is not possible to determine both the fever focus and the infectious agent during the initial phase. In these cases, infection markers are frequently used as a means of determining the initiation of antibiotic treatment or the necessity of hospitalization (7). Acute phase reactants are used when clinical findings are insufficient to distinguish bacterial infection in febrile cases. The most commonly applied reactants for this purpose are leukocyte count, neutrophil (NEU) count or neutrophil–lymphocyte (LYM) ratio (NLR), platelet count, erythrocyte sedimentation rate (ESR), and C-reactive protein (CRP) (8–10). However, it has been suggested that the reactants mentioned are not specific in detecting bacterial infection in some cases, hence necessitating the identification of novel markers.

In most cases, the presence of leukocytosis and/or increased CRP levels (>20 mg/L) and high ESR (>25 mm/h) are observed (11). In recent studies on inflammatory diseases, NLR and platelet–lymphocyte ratio (PLR) have been found to be associated with disease activity (11–13). Systemic immune-inflammation index (SII) is calculated by utilizing platelet, neutrophil, and lymphocyte counts together. Compared with PLR and NLR, it is a much more important marker in indicating inflammation and immune response. Studies have shown that high SII values are associated with the severity of the disease and poor prognosis in many diseases and malignancies (14).

Accordingly, the aim of this study is to determine whether leukocytes [white blood cell (WBC)], CRP, NLR, PLR, and SII are useful markers to predict urine culture growth in pediatric patients with UTI. Second, the aim of this study is to evaluate the prevalence of UTI pathogens, antibiotic resistance patterns, and empirical treatment options in children diagnosed with UTI based on laboratory and clinical findings.

## Material methods

### Participants and procedures

The ethical approval for this study was obtained from the non-interventional ethics committee of the Medical Faculty of Istanbul Atlas University (No: E-22686390-050.01.04-14258, Date: 13 January 2023). The study was performed in accordance with the Helsinki Declaration. Informed consent was obtained from all subjects prior to enrollment.

**Figure 1:**
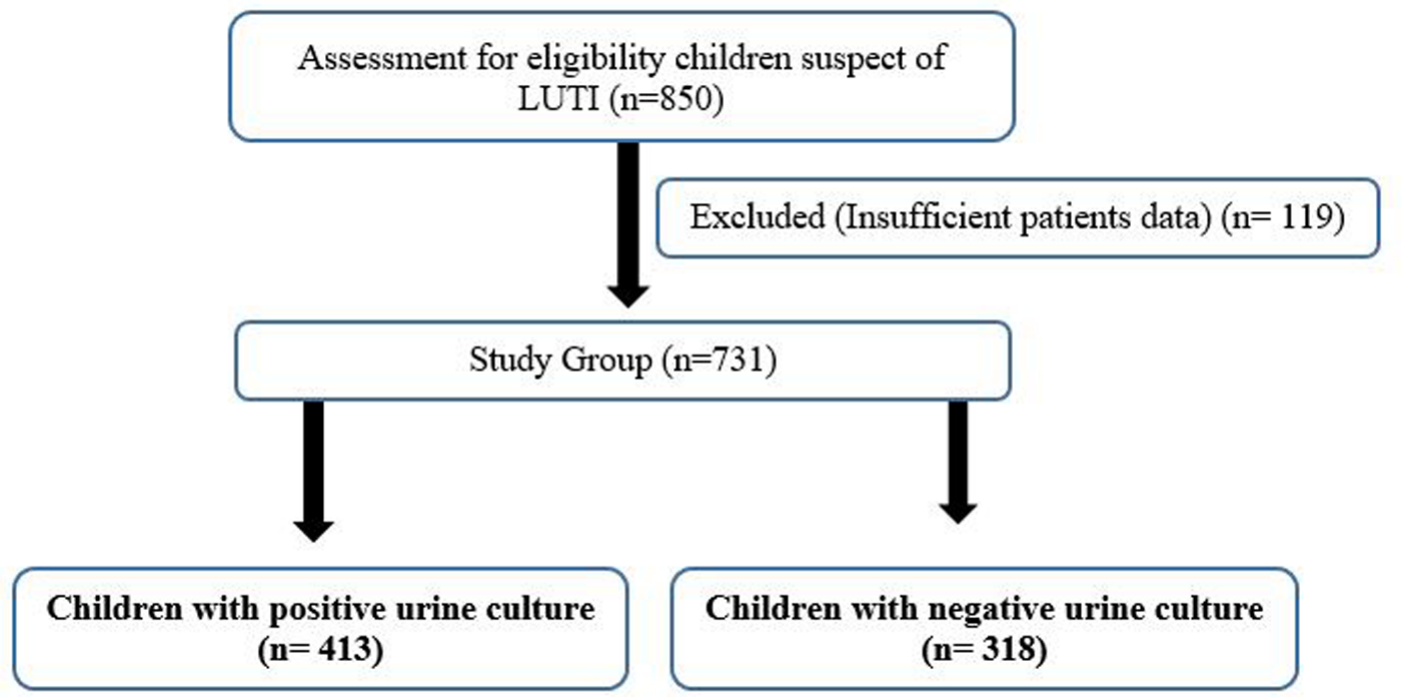
Flowchart of children with lower urinary tract infection (UTI).

This study evaluated 413 patients (positive urine cultures) and 318 patients (negative urine cultures) who presented to the pediatric emergency clinic of Istanbul Atlas University-Medical Faculty-Medicine Hospital with UTI complaints and diagnosis of UTI between July 2021 and June 2022. The flowchart of children with UTI is shown in Figure 1. Data on the gender, age, antibiotic resistance, and laboratory results of patients aged 3–18 years were diagnosed with UTI according to international guidelines (15) and were followed up for at least 1 year were obtained from their medical records.

The microbiology unit collected the urine culture samples, specifically targeting clean and mid-stream urine specimens. The obtained cultures were identified according to the Clinical & Laboratory Standards Institute (CLSI) criteria (16). Urinalysis with pyuria [WBC > 5/HPF (white blood cells per high power field (WBC/HPF)] and urine culture with >50,000 CFU/ml pathogenic organism were included in the study. Patients with known chronic diseases (chronic kidney disease), congenital renal anomalies, urinary tract abnormality, neurogenic bladder, renal calculi, other bacterial infection, and immune deficiency were excluded from the study.

Trimethoprim–sulfamethoxazole (TMP-SMX) or cephalosporins (cefadroxil, cephalexin, cefuroxime, cefixime) were generally used as the first choice for antimicrobial treatment prior to culture. A urine culture taken 48 h later revealed sensitivity to specific antibiotics, and the antibiotic selection was determined based on the patient's clinical condition. The patients were monitored by different physicians. The clinical decision regarding antibiotic use, including the specific antibiotic chosen and its duration, was made by the physician. Based on the patient records in our study, it was observed that physicians mostly prescribed antibiotics for 10 days. Among the culture positive group, 357 patients (86.4%) received antibiotics for 10 days, 13 patients (3.1%) received antibiotics for 8 days, 38 patients (9.2%) for 7 days, and three patients (0.7%) for 5 days. In addition, two patients (0.5%) were given antibiotics, but the duration was not recorded (missing data). No antibiotics were administered for more than 10 days.

The antibiotic treatment regimen of children with UTI under treatment is given in Table 3.

The demographic and clinical characteristics of the cases included in the study were determined retrospectively from the file records. The age of complaint onset (days), age at admission (days), duration of symptoms (days), gender, complaint, gestational age (weeks), nutrition (breast milk), physical examination findings, laboratory results, antibiotic use, detected causative virus, administered treatments, and length of stay (days) were collected from the electronic patient care records.

### Measures

Microorganisms were determined using the VITEK 2 (bioMerieux) automated system, and an antibiogram was performed to identify susceptibility patterns.

Serum CRP levels were measured nephelometrically (Siemens-Dimention, Germany). The complete blood count (CBC) was measured using an automatic hematology analyzer (Sysmeks XN-1000, Germany). NLR and PLR are calculated from the peripheral blood sample as the ratio of neutrophil and platelet count to lymphocyte count. The SII was calculated as (platelet count ×  neutrophil count)/lymphocyte count (17).

### Statistical analysis

IBM SPSS (The Statistical Package for the Social Sciences) version 21.0 package program was used for the evaluation and analysis of the data. Descriptive data were expressed as frequency (*n*) and percent (%) for categorical variables, and as mean ± standard deviation or median (25th percentile–75th percentile) for numerical variables. The normality of continuous variables was evaluated using the Kolmogorov–Smirnov test. Chi-square test was used to compare categorical variables. To compare continuous variables between two independent groups, the independent group *t*-test was used if normal distribution is provided; if normal distribution was not achieved, Mann–Whitney *U*-test was used. *P* < 0.05 was accepted for statistical significance.

## Results

**Figure 2:**
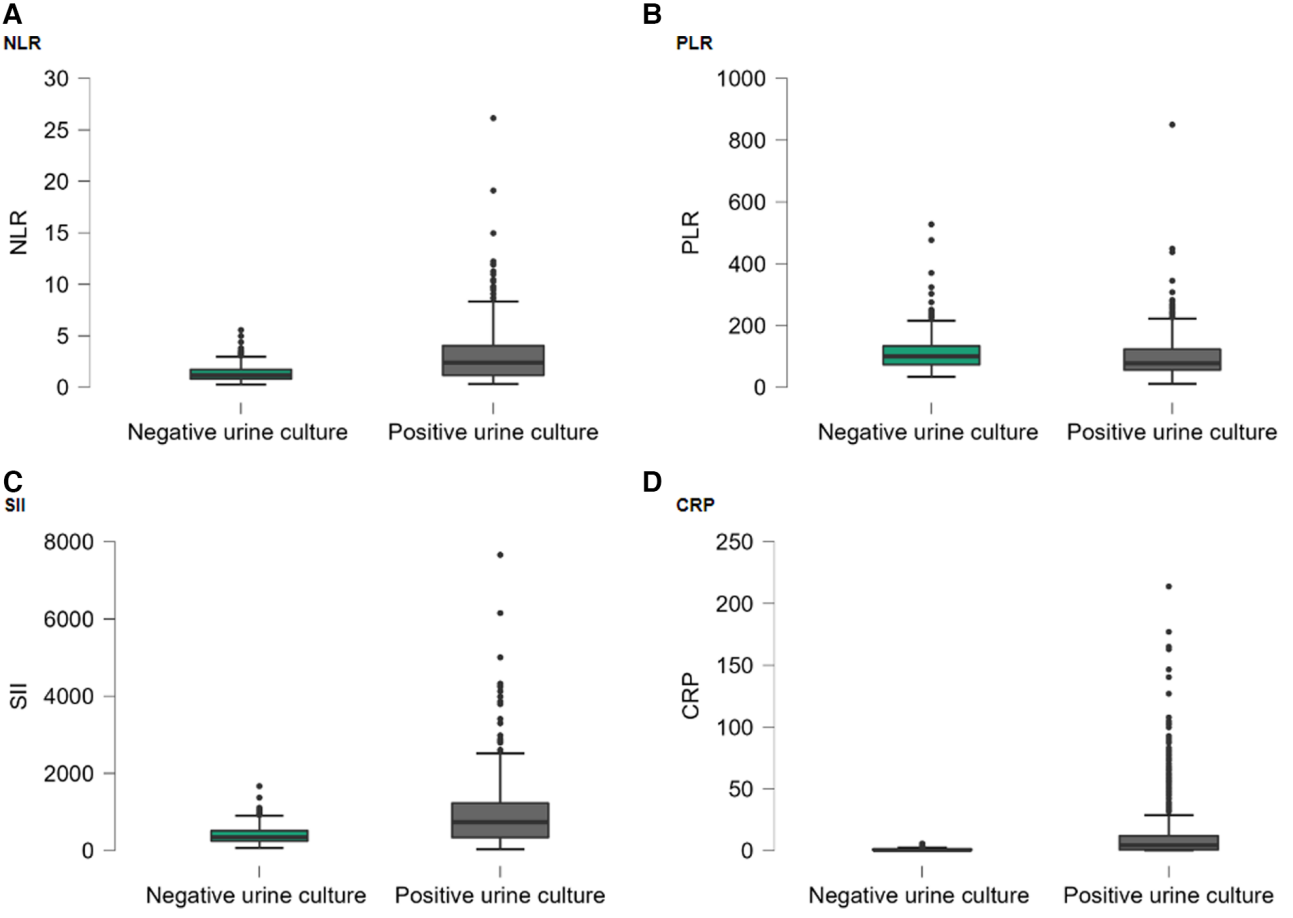
Box-plot graph by hospitalization groups: (**A**) NLR; (**B**) PLR; (**C**) SII; (**D**) CRP. Points (●) represent the outliers.

The demographic and clinical characteristics of the cases included in the study are shown in Table 1. The gender and age distribution were similar between groups. The prevailing symptoms observed included burning in urine, fever, and abdominal pain. The laboratory findings of the included cases are shown in Table 2. While the median WBC value of the positive urine culture group was 8.1 (6.5–10.9), it was 7.0 (6.0–8.1) in the negative urine culture group. There was a statistically significant difference found between groups. There were no statistically significant differences observed in the medians of hemoglobin (HGB), hematocrit (HCT), and platelet (PLT) between the two groups. The median of lymphocytes, neutrophil, and monocytes (MONO) of the positive urine culture group was statistically significantly higher than that of the negative urine culture group. The median NLR was 2.4 (1.17–4.04) in the positive urine culture group and 1.18 (0.83–1.71) in the negative urine culture group. The median PLR was 77.53 (55.98–123.22) in the positive urine culture group and 100.11 (73.35–133.43) in the negative urine culture group. NLR was statistically significantly higher and PLR was statistically lower in the positive urine culture group (*p* < 0.001; *p* < 0.001) compared with that of the negative culture group. The median of SII was significantly higher in the positive urine culture group than that of the negative urine culture group [736 (343–1,230); 344 (247–515); *p* < 0.001]. In addition, the median of CRP was significantly higher in the positive urine culture group than that of the negative urine culture group [4.41(0.67–11.97); 0.6(0.27–1.13); *p* < 0.001] (Figure 2). It was observed that the positive urine culture group received peroral antibiotics for 10 days, and the median number of bacteria colonies was 100,000.

**Table 1:** Demographic and clinical characteristics of the cases included in the study.

	Negative urine culture group		Positive urine culture group		*p*-value
	*n*	%	*n*	%	
Gender					
Boy	37	11.6%	45	10.9%	0.754^a^
Girl	281	88.4%	368	89.1%	
Age (years)	6 (4–8)		6 (4–8)		**0.393** ^b^
Symptoms					
Fever	85	26.7%	111	26.9%	0.965^a^
Abdominal pain	118	37.1%	92	22.3%	**<0**.**001**^a^
Frequent urination	40	12.6%	67	16.2%	0.167^a^
Odor in urine	8	2.5%	26	6.3%	**0**.**016**^a^
Groin pain	19	6.0%	60	14.5%	**<0**.**001**^a^
Color change in urine	19	6.0%	36	8.7%	0.164^a^
Burning in urine	139	43.7%	249	60.3%	**<0**.**001**^a^
Vomiting	74	23.3%	40	9.7%	**<0**.**001**^a^
Constipation	6	1.9%	13	3.1%	0.288^a^
Hospitalization					
No	318	100%	403	97.6%	
Yes	0	0%	10	2.4%	**0**.**003**^b^
The length of hospitalization	—		10.6 (7.7–14.1)		—

Bold *p* values indicate *p* < 0.05.

^a^ Chi-square test.

^b^ Mann–Whitney *U*-test.

^c^ Fishers exact test was applied.

**Table 2:** Laboratory characteristics of the cases included in the study.

	Negative urine culture group	Positive urine culture group	*p*-value
WBC (×10^3^/µl)	7.0 (6.0–8.1)	8.1 (6.5–10.9)	**<0**.**001**^a^
HGB (g/dl)	12.4 (11.7–13)	12.4 (11.7–13.03)	0.866^b^
HCT (%)	36.8 (34.6–38.7)	36.95 (35–38.63)	0.661^b^
PLT (×10^3^/ml)	289 (259–330)	303 (245–357)	0.079^a^
LYM (10^3^/µl)	2.9 (2.24–3.96)	3.88 (2.71–5.2)	**<0**.**001**^a^
NEU (10^3^/µl)	3.55 (2.77–4.5)	8.13 (4.39–13.43)	**<0**.**001**^a^
MONO (10^3^/µl)	0.73 (0.51–1.1)	0.84 (0.6–1.3)	**0**.**015**^a^
NLR	1.18 (0.83–1.71)	2.40 (1.17–4.04)	**<0**.**001**^a^
PLR	100 (73–133)	78 (56–123)	**<0**.**001**^a^
SII	344 (247–515)	736 (343–1,230)	**<0**.**001**^a^
CRP (mg/l)	0.6 (0.27–1.13)	4.41 (0.67–11.97)	**<0**.**001**^a^
Number of colonies (CFU/ml)	–	100,000 (50,000–100,000)	–
The length of using antibiotics (day)	–	10 (10–10)	–

Bold *p* values indicate *p* < 0.05.

^a^ Mann–Whitney *U*-test used.

^b^ Independent group *t*-test.

The microorganisms reproduced in the positive urine culture group, the bacteria to which they are resistant, and the antibiotics they receive are shown in Table 3. Accordingly, it was observed that the most prevalent microorganisms identified were *E.coli*, accounting for 77% of the total, followed by *Klebsiella pneumoniae* at 7.3%, *Proteus vulgaris* at 4.8%, *Proteus* spp. at 3.9%, and *Pseudomonas aeruginosa* at 2.2%.

**Table 3:** Microorganisms grown, resistant to antibiotics, and antibiotics used in the positive urine culture group.

	*n*	%
Reproducing microorganism		
*E. coli*	318	77.0
*Klebsiella pneumoniae*	30	7.3
*Proteus vulgaris*	20	4.8
*Proteus* spp.	16	3.9
*Pseudomonas aeruginosa*	9	2.2
*Enterococcus* spp.	4	1.0
*Candida* spp.	3	0.7
*Acinetobacter* spp.	2	0.5
*Staphylococcus aureus*	2	0.5
*Staphylococcus epidermidis*	2	0.5
*E. coli- proteus vulgaris*	1	0.2
*Enterobacter* spp.	1	0.2
*Coagulase negative (-) staphylococcus*	1	0.2
*Morganella morganii*	1	0.2
*MRSA*	1	0.2
*Staphylococcus epidermidis (meticilline)*	1	0.2
*Staphylococcus simulans*	1	0.2
Resistant to antibiotics		
Cefuroxime	127	30.8
Trimethoprim–sulfamethoxazole	117	28.3
Cefixime	112	27.1
Ampicillin	103	24.9
Ceftriaxone	88	21.3
Cefotaxime	86	20.8
Nitrofurantoin	64	15.5
Cefepime	34	8.2
Fosfomycin	28	6.8
Ampicillin–sulbactam	27	6.5
Ciprofloxacin	24	5.8
Ceftazidime	22	5.3
Cefazoline	14	3.3
Gentamicin	9	2.2
Tigecycline	9	2.2
Ertapenem	8	1.9
Ofloxacin	8	1.9
Piperacillin–tazob	7	1.7
Amoxicillin–clavulanic acid	6	1.5
Levofloxacin	5	1.2
Cefoperazone/sulbactam	5	1.2
Amikacin	5	1.2
Cefoxitin	5	1.2
Bactrim	4	1.0
Tetracycline	3	0.7
İmipenem	3	0.7
Meropenem	2	0.5
Clindamycin	2	0.5
Erythromycin	1	0.2
Penicillin	1	0.2
Oxacillin	1	0.2
Rifampicin	1	0.2
Linezolid	1	0.2
Tobramycin	1	0.2
Antibiotic used		
Cephalosporin	378	91.5
Penicillin	10	2.4
Nitrofurantoin	8	1.9
Trimethoprim + sulfamethoxazole	6	1.5
Aminoglycoside	4	1.0
Trimethoprim	4	1.0
Quinolone	1	0.2
Nitrofurantoin, trimethoprim	1	0.2
Cephalosporin, ceftriaxone	1	0.2

The microorganisms in the positive urine culture group had the highest frequency of reproduction with cefuroxime at 30.8%, TMP-SMX at 28.3%, cefixime at 27.1%, ampicillin at 24.9%, and ceftriaxone at 21.3%.

The most frequently used antibiotics in the urine positive culture group were the cephalosporin group with 91.5%, followed by penicillin with 2.4% and nitrofurantoin with 1.9%.

Table 4 shows the correlation analysis results between WBC, CRP, NLR, PLR, and SII. CRP was weakly correlated with NLR in the negative urine culture group and all groups. CRP was weakly correlated with PLR in the negative urine culture group and positive urine culture group, although it was not correlated in all groups.

**Table 4:** The results of Spearman's correlation analyses between inflammatory markers and indexes in all groups and sub-groups.

	All Groups		Negative urine culture group		Positive urine culture group	
	*r*	*p*	*r*	*p*	*r*	*p*
WBC-CRP	0.512	<0.001	−0.017	0.776	0.475	<0.001
WBC-NLR	0.273	<0.001	0.008	0.887	0.183	<0.001
WBC-PLR	0.047	0.209	0.125	0.025	0.085	0.085
WBC-SII	0.252	<0.001	0.001	0.979	0.158	<0.001
CRP-NLR	0.113	<0.001	0.144	0.010	−0.004	0.930
CRP-PLR	0.048	0.74	0.186	0.001	0.103	0.037
CRP-SII	0.081	<0.001	0.114	0.042	−0.041	0.410

Spearman correlation test used.

**Figure 3:**
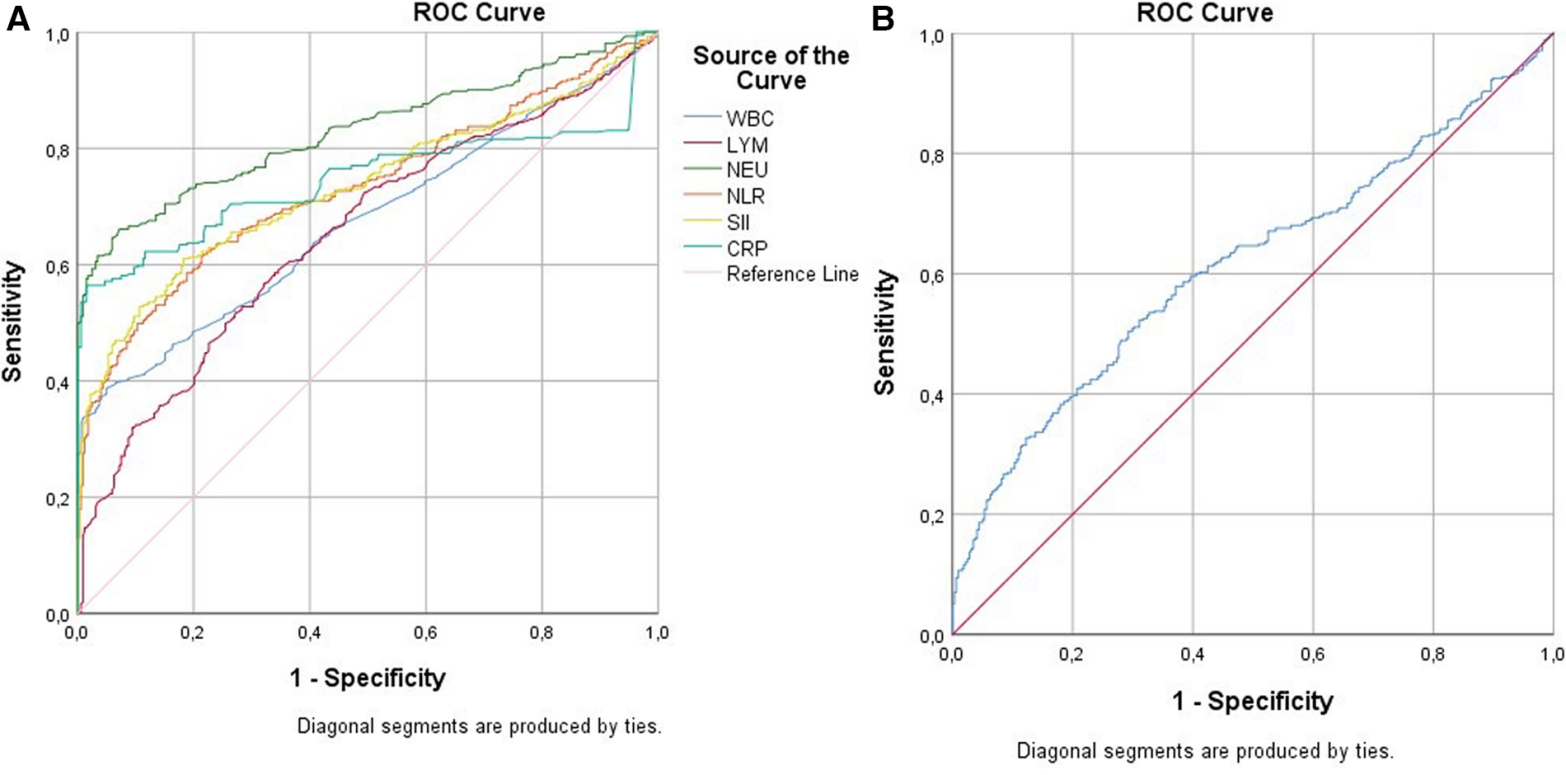
(**A**) WBC, LYM, NEU, NLR, SII, CRP; (**B**) PLR.

Table 5 and Figure 3 represent the ROC analysis results for WBC, LYM, neutrophil, NLR, SII, CRP, and PLR in terms of UTI. For a cutoff value of 10 of WBC, the sensitivity was found to be 33.4%, and the specificity was 99.1%. For a cutoff value of 6.4 of WBC, the sensitivity was 76.8%, and the specificity was 35.1%. For a cutoff value of 3.5 of LYM, the sensitivity was 57.6%, and specificity was 66.7%. For a cutoff value of 6 of neutrophil, the sensitivity was 64.4%, and the specificity was 94.0%. For a cutoff value of 2 of NLR, the sensitivity was 57.4%, and the specificity was 81.1%. For a cutoff value of 600 of SII, the sensitivity was 58.4%, and the specificity was 83.0%. For a cutoff value of 3.2 of CRP, the sensitivity was 56.4%, and the specificity was 98.4%. For a cutoff value of 80 of PLR, the sensitivity was 52.5%, and the specificity was 68.2%.

**Table 5:** The results of ROC analyses for markers in terms of lower urinary tract infection.

Parameter	AUC	95% CI	*p*-value	Cutoff	Sensitivity	Specificity	Accuracy
WBC	0.674	0.636–0.712	<0.001	10^b^	33.4%	99.1%	62.0%
LYM	0.653	0.614–0.693	<0.001	3.5^b^	57.6%	66.7%	61.6%
NEU	0.829	0.8–0.859	<0.001	6^b^	64.4%	94.0%	77.3%
NLR	0.732	0.697–0.769	<0.001	2^b^	57.4%	81.1%	67.7%
SII	0.733	0.697–0.769	<0.001	600^b^	58.4%	83.0%	69.1%
CRP	0.747	0.71–0.784	<0.001	3.2^b^	56.4%	98.4%	74.7%
PLR	0.615	0.575–0.655	<0.001	80^a^	52.5%	68.2%	59.4

^a^ If less than or equal to.

^b^ If greater than or equal to.

## Discussion

In this study, we investigated the relationship between new generation systemic immune-inflammation index parameters and UTI in children. To the best of our knowledge, this is the first study in which the NLR, PLR, SII, and CRP was used to predict the presence of pathogens in UTI. We also found that a CRP of more than 3.20 predicted the presence of pathogens in UTI with a sensitivity of 56.4% and a specificity of 98.4% with 74.7% accuracy. Although CRP, NLR, PLR, and SII have high values, CRP is a better marker to exclude culture positivity due to its higher specificity.

Children older than 2 years of age present with abdominal pain and fever, and school-age children present mostly with symptoms of lower urinary tract infection such as dysuria, suprapubic tenderness, sudden and frequent urination, and complaints such as abdominal pain, flank pain, voiding disorders, constipation, and fever. In the present study, abdominal pain has been found in 37.1% of patients in the negative urine culture and in 22.3% of patients in the positive urine culture. Abdominal pain in childhood can often be seen without any disease, and therefore it is relative. Symptoms in children may include frequent urination, newly occurring bedwetting, difficulty urinating, and abdominal pain after urination. However, there was no difference observed between the groups regarding symptoms of fever, frequency of urination, and color change in urine. While abdominal pain and vomiting symptoms are higher in the negative urine culture group, symptoms such as odor in urine, groin pain, burning in urine were higher in the positive urine culture group. Odor in urine, groin pain, and burning in urine can be seen as more clinically severe cases, so the frequency of being positive may be high. Abdominal pain and vomiting were non-specific, and these were the more frequently reported symptoms. Therefore, it may be high in the negative urine culture group.

It is important that UTI shows a wide spectrum from asymptomatic bacteriuria to symptomatic complicated pyelonephritis in children and that the clinical evaluation of the patient is performed correctly in terms of complications that may occur in the future (18). *E. coli* is the bacterium that most commonly causes urinary infection. Based on the results of our study, the most commonly grown microorganism was *E. coli* (77%), which is consistent with the literature (19). In family medicine and pediatric clinics, which are usually the first place of referral of patients, specifically in cases where urine culture cannot be performed, tests supporting the culture can help to start empirical treatment correctly. To prevent complications, UTI should be treated empirically until the urine culture is concluded. However, antibiotic resistance against uropathogens is increasing worldwide due to inappropriate antibiotic use and failure of empirical treatment in community acquired UTIs. This situation reduces the effect of antibiotics in the treatment and prophylaxis of UTI (20–22). The choice of antibiotic should be based on local epidemiology and susceptibility patterns (23). In the present study, abdominal pain, odor in urine, groin pain, burning in urine, and vomiting were significantly higher in grown-positive patients than in grown-negative patients. The microorganisms in the positive urine culture group had the highest frequency of reproduction with cefuroxime at 30.8%, TMP-SMX at 28.3%, cefixime at 27.1%, ampicillin at 24.9%, and ceftriaxone at 21.3%. The positive urine culture group used oral antibiotics for 10 days, and the median number of bacteria colonies was 100,000. The most frequently used antibiotics in the positive urine culture group were the cephalosporin group with 91.5%, followed by penicillin with 2.4% and nitrofurantoin with 1.9%. The microorganisms were resistant to cefuroxime at most 30.8%, trimethoprim–sulfamethoxazole 28.3%, cefixime at 27.1%, ampicillin at 24.9%, ceftriaxone at 21.3%, cefotaxime at 20.8%. *E. coli* is the most common problem. While both tigecycline and ertapenem were susceptible to 99.37%, the microorganisms were resistant to cefuroxime at 31.76%, trimethoprim–sulfamethoxazole at 27.04%, cefixime at 28.62%, and ampicillin at 22.01%. The findings of the study conducted by Salduz et al. (24) found ampicillin resistance at 100%. *E. coli* was highly resistant to ampicillin, while third-generation cephalosporins exhibited greater *in vitro* efficacy. Implementing antimicrobial stewardship programs and regular monitoring of antimicrobial resistance could help to minimize inappropriate medication for UTIs (25).

In order to apply the right treatment, it is very important to determine the causative bacteria correctly and to use auxiliary tests where culture is not possible. The growth of significant bacteria in urine samples taken under appropriate conditions is the gold standard for the diagnosis of UTI. However, it is an expensive test, results take a long time, and not available in every center (26). Although not directly used for the diagnosis of UTI, there are studies showing that acute phase markers are associated with UTI in recent years (27). CBC is a widely used diagnostic tool because of its cost-effectiveness, fast, and frequently reproducible features, as well as the data diversity it offers. In the present study, the numbers of WBC, LYM, NEU, and MONO were significantly higher in the growth-positive patients. WBC count and difference were used as predictive markers in the studies (28). As a result, leukocytosis can be seen in the course of UTI. In children with UTI, it is believed that clinical evaluation of symptoms and complaints, followed by the examination of hematological parameters, can be used as inflammatory markers.

UTIs are difficult to diagnose in young children. The most important difficulties are the absence of specific findings at this age and the difficulty to collect uncontaminated urine samples without using invasive methods such as urethral catheterization and suprapubic aspiration. However, since UTI is accompanied by specific symptoms after infancy, it is easily diagnosed and treated (29).

The NLR, PLR, and SII have emerged as topics of significant interest in recent academic research. These parameters, which are considered as markers of inflammation, have also been shown to be prognostic indicators in critically ill septic patients and allergic rhinitis in different studies (30, 31). Asik (32) reported that since NLR and PLR were higher in patients with urinary tract infections compared with the healthy volunteer control group, initially, the patient's symptoms and complaints should be evaluated clinically, and then NLR and PLR could be used as inflammatory markers. Han et al. (11) showed that NLR was a predictive factor for positive Tc^99m^ DMSA scintigraphy (DMSA) defects, surpassing the efficacy of traditional markers for predicting vesicoureteral reflux (VUR). SII, CRP, PLR, and NLR have a predictive ability to discriminate renal involvement from normal renal findings in newborns with UTI. SII might be used as an additional parameter in the prediction of renal involvement in newborns with UTIs (11). Lee et al. (33) found that NLR was significantly associated with persistent cortical defect on the follow-up DMSA scan and positive cortical defect on the initial scan in patients without VUR in children with febrile UTI. In the present study, NLR and SII were higher and PLR was lower in the positive urine culture group than the negative urine culture group. These parameters can be useful in differentiating positive and negative urine cultures in an algorithm used to diagnose UTI.

CRP, which is generally elevated in acute bacterial infections, is an acute phase reactant that is frequently used (34). CRP also represents biomarkers with potential to aid the clinician in the diagnosis and management of UTI (35). Gervaix et al. (36) showed that a rapid determination of procalcitonin (PCT) concentration from the CRP concentration could be more useful for the management of children with febrile UTI in the emergency room. In another study, the combination of four variables as fever peak of >39°C, urinary bladder sonography, PCT, and CRP had the highest power in predicting APN in children with UTI (37). In the meta-analysis, CRP and PCT have low accuracy for cystitis, but might be useful for PN (38). Both PCT and CRP can be used for UUTI and LUTI differentiation, but PCT has higher sensitivity and specificity in predicting PN than CRP (39). The AUC values for CRP were higher than those of other inflammatory markers such as WBC, ESR, and MPV levels for APN in UTI. In line with our findings, platelet count did not differ between children with APN and those with LUTI (42). NLR in CBC is a novel and useful biomarker for predicting acute kidney injury in patients with UTI (40). Soyaltın et al. (41) evaluated the predictability of WBC, platelet counts, MPV, NLR, and PLR to predict UTIs caused by extended-spectrum beta-lactamase-producing bacteria (ESBL-PB) in infants. On the contrary, they could not demonstrate that the inflammation markers were reliable markers for the prediction of ESBL-PB positivity (41). In the present study, the median of CRP was significantly higher in the positive urine culture group than the positive urine culture group. CRP was weakly correlated with NLR in the negative urine culture group and all groups. CRP was weakly correlated with PLR in the negative urine culture group and the positive urine culture group. Although the specificity of CRP is higher than NLR and SII, NLR and SII are also powerful parameters in predicting inflammatory status and unnecessary antibiotic use. An accurate diagnosis of UTI is essential in children. There is a risk of recurrence of UTIs in 20%–30% of boys and 40%–60% of girls after the initial infection. In addition to other markers, we believe that the combination of NLR, PLR, SII, and CRP are useful in determining the prediction of pediatric patients with UTI who have positive urine culture in addition to other parameters until the urine culture results are identified.

### Study limitations

The present study had several limitations. First, the study is retrospective in nature and was conducted in a single center. The second limitation is the start of the regular use of antibiotics. Third, the absence of procalcitonin levels as a means of distinguishing between bacterial and viral infections is another limitation of the study. Finally, it is important to note that the study did not include children with other bacterial infections, such as bacteremia, or those who were experiencing inflammatory conditions, such as Kawasaki disease.

The results of the study showed that the issue of increasing antibiotic resistance remains a significant concern in UTIs. *E. coli* was identified as the most common causative agent, and the most commonly prescribed antibiotic was cephalosporin. However, it was observed that all identified agents of pediatric UTIs in our center exhibited high resistance to cefuroxime, TMP-SMX, cefixime, ampicillin, and ceftriaxone. WBC, CRP, NLR, PLR, and SII could potentially serve as useful independent diagnostic or complementary markers for disease in children diagnosed with UTI who have positive urine culture. Although CRP, NLR, PLR, and SII exhibited elevated levels, CRP is a more effective marker to exclude culture positivity due to its higher specificity. However, both NLR and SII are also powerful parameters in predicting inflammatory status and unnecessary antibiotic use. The combination of NLR, PLR, SII, and CRP appears to be a useful prognostic predictor for urine culture outcomes in pediatric patients with UTI. Further studies are needed to validate our results.

## Data Availability

The original contributions presented in the study are included in the article/Supplementary Material, further inquiries can be directed to the corresponding author.
